# The essential role of YAP in ERα36-mediated proliferation and the epithelial-mesenchymal transition in MCF-7 breast cancer cells

**DOI:** 10.3389/fphar.2022.1057276

**Published:** 2022-12-02

**Authors:** Miso Park, Seung Hyun Lee, Quyen Thu Bui, Young-Mi Kim, Keon Wook Kang

**Affiliations:** ^1^ College of Pharmacy and Research Institute of Pharmaceutical Sciences, Seoul National University, Seoul, South Korea; ^2^ Department of Pharmacy and Institute of Pharmaceutical Science and Technology, Hanyang University, Ansan, South Korea

**Keywords:** breast cancer, Yap (Hippo) signaling, src, MCF- 7 cancer cell line, ERα36

## Abstract

**Purpose:** Most breast cancers are hormone-receptor-positive, and thus the first-line therapy for them is an anti-estrogen medication such as tamoxifen. If metastasis occurs or resistance to tamoxifen develops, the 5-year survival rates for breast cancer patients significantly decrease. Hence, a better understanding of the molecular mechanisms that contribute to breast cancer aggressiveness is of great importance. ERα36 is an estrogen receptor variant that is known to be upregulated in breast cancer patients receiving tamoxifen treatment or in triple-negative breast cancer cells. However, the specific molecular mechanism underlying ERα36-induced tamoxifen-resistance is not yet fully understood.

**Methods:** ERα36-overexpressing MCF-7 cells were constructed by either plasmid transfection using ERα36 vector or retroviral infection using ERα36-V5-His vector. Target-gene expression was assessed by Western blot analysis and real-time PCR, and YAP activation was evaluated by luciferase assays and immunofluorescence. Cell proliferation and formation of three-dimensional spheroids were evaluated using the IncuCyte S3 Live Cell Analysis System.

**Results:** We found that the expression patterns of Hippo signaling-related genes were significantly changed in ERα36-overexpressing MCF-7 cells compared to MCF-7 cells, which were also similarly observed in tamoxifen-resistant MCF-7 cells. Specifically, the protein expression level and activity of YAP, the core downstream protein of the Hippo pathway, were significantly increased in ERα36-overexpressing MCF-7 cells compared with MCF-7 cells. The aggressive phenotypes acquired by ERα36 overexpression in MCF-7 cells were destroyed by YAP knockout. On this basis, we propose that ERα36 regulates YAP activity by a new mechanism involving Src kinase.

**Conclusion:** Our results suggest that YAP targeting may be a new therapeutic approach to the treatment of advanced breast cancers overexpressing ERα36.

## Graphical Abstract

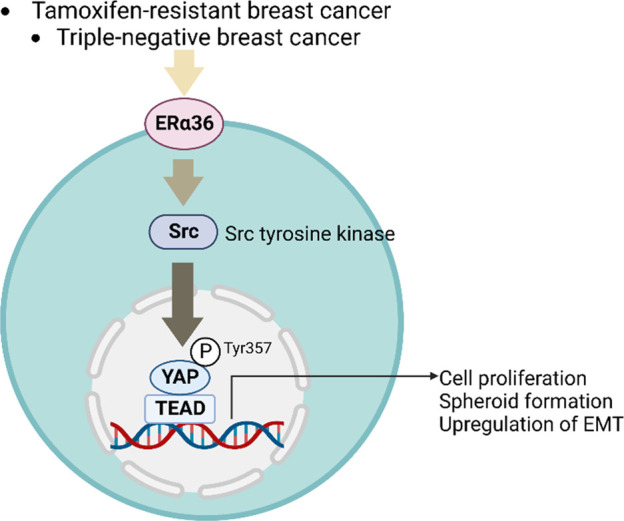

## Highlights

•ER36 overexpression in MCF-7 cells increases the expression of EMT-related genes and destroys the estrogen dependency of MCF-7 cells for cell proliferation.
•YAP is highly activated in ER36-overexpressing MCF-7 cells.
•YAP knockout inhibits proliferation and expression of EMT-related genes in ER36-overexpressing MCF-7 cells.
•Src activation is responsible for ER36-mediated YAP activation.

## Introduction

Breast cancer is the most common cancer among women. In the United States, it is the second most common cause of cancer-related death among women (Siegel et al., 2021). The growth of breast cancer highly depends on estrogen signaling, 70% of cases being estrogen receptor (ER) α-positive (Onitilo et al., 2009). Anti-estrogen therapy such as tamoxifen is the first-line treatment for ER-positive breast cancer patients. However, as most patients eventually become resistant to tamoxifen, there is still an urgent need to find novel pathways for new therapeutic strategies.

Estrogen receptors ERα and ERβ mediate the biological effects of estrogen. The estrogen receptor α36 (ERα36), first identified by Wang et al., in 2005 (Wang et al., 2005), is a transcriptional variant of 66 -kDa ERα that lacks the transcriptionally active domains AF-1 and AF-2 but retains the DNA-binding domain along with some ligand-binding domains (Wang et al., 2005). Since the discovery of ERα36, a number of studies have extensively explored the functions of this variant receptor and their underlying molecular mechanisms (Wang and Yin, 2015; Mahboobifard et al., 2021). For example, in tamoxifen-resistant breast cancer cells as well as triple-negative breast cancer (TNBC) cells, the level of ERα36 was increased while that of ERα66 disappeared in those cell lines (Zhang et al., 2012; Li et al., 2013; Maczis et al., 2018). Furthermore, overexpression of ERα36 was found to contribute to acquisition of tamoxifen resistance (Zhang and Wang, 2013; Wang and Yin, 2015). For patients whose tumors showed expression of both ERα36 and ERα66, tamoxifen therapy was less effective (Shi et al., 2009). In another study, tamoxifen could activate ERα36 for upregulation of aldehyde dehydrogenase 1A1 expression, which increased the stemness and metastasis of breast cancer cells (Wang et al., 2018). However, the specific molecular mechanism(s) underlying ERα36-induced tamoxifen-resistance has yet to be fully elucidated.

The Yes-associated protein (YAP or YAP1) is the downstream core protein in the Hippo signaling pathway. YAP has gained considerable interest due to its functions as a potent tumor promoter and its frequent activation across multiple tumor types (Zanconato et al., 2016). It is a transcriptional coactivator that shuttles between the cytoplasm and the nucleus. In the nucleus, YAP interacts with other transcription factors, particularly members of the TEA domain (TEAD) family, which upregulates target-gene expression including cysteine-rich 61 (CYR61) and connective tissue growth factor (CTGF), both of which are associated with cancer development, progression, and metastasis (Piccolo et al., 2014). Dephosphorylation or phosphorylation of particular residues on YAP can regulate nuclear translocation and promote cell proliferation by interacting with multiple transcription factors (Piccolo et al., 2014; Dasgupta and McCollum, 2019). Abnormal regulation of YAP drives key aspects of the epithelial-to-mesenchymal transition (EMT), which are crucial for cancer stemness and metastasis (Zhao et al., 2010; Cordenonsi et al., 2011). In particular, many studies have found that YAP activation empowers cancer cell to resist chemotherapies and targeted anticancer therapies including rapidly accelerated fibrosarcoma (RAF), mitogen-activated protein kinase (MEK), and human epidermal growth factor receptor 2 (HER2) inhibitors (Lin et al., 2015; Kim et al., 2016; Zanconato et al., 2016). Hence, identification of upstream regulatory signaling of YAP may contribute to the establishment of new therapeutic strategies.

Beyond the crucial roles of ERα36 in breast cancer, there remains a gap in our understanding of how ERα36 can mediate breast cancer cell aggressiveness. To fill this knowledge gap, in the present study, we constructed an ERα36-overexpressing MCF-7 (MCF-7-ERα36) cell line and performed a transcriptomic analysis in ER-positive MCF-7, MCF-7-ERα36, and tamoxifen-resistant MCF-7 (TAMR-MCF-7) cells. We found the expression pattern of genes involved in the Hippo pathway to be very similar between the TAMR-MCF-7 and MCF-7-ERα36 cells. According to our results, herein we will demonstrate that YAP is involved with the aggressive phenotypes of MCF-7-ERα36 cells, specifically by increasing cell proliferation and the 3D spheroid volume and acquiring EMT phenotypes. Our study also indicates that, in mechanistic terms, activation of non-canonical Src signaling is responsible for ERα36-induced YAP activation.

## Materials and methods

### Cell culture

MCF-7 and MCF-7-ERα36 cells were cultured in Dulbecco’s modified Eagle’s medium (DMEM) supplemented with 10% fetal bovine serum (FBS) and penicillin/streptomycin. TAMR-MCF-7 cells were cultured in DMEM supplemented with 10% charcoal stripped FBS (Gemini Bio Product, CA, USA), penicillin/streptomycin and 3 μM (Z)-4-hydroxytamoxifen (Tocris Bioscience, Bristol, United Kingdom). TAMR-MCF-7 cells were established as previously reported (Park et al., 2019).

### Reagents

Verteporfin (#SML0534) and antibodies recognizing β-actin (#a2228), FLAG^®^ M2 (#F1804), glyceraldeyde-3-phosphate dehydrogenase (GAPDH, #CB1001) and 17-β-estradiol (#E1024) were purchased from Merck (Billerica, MA, USA). Antibodies recognizing YAP/TAZ (#8418), p-YAP (Ser127) (#4911), zinc finger E-box binding homeobox1 (ZEB1, #3396), Src (#2108), p-Src (Tyr416) (#2101), horseradish peroxidase-conjugated donkey anti-rabbit IgG (#7074), and horseradish peroxidase-conjugated donkey anti-mouse IgG (#7076) were purchased from Cell Signaling Technology (Danvers, MA, USA). Antibodies recognizing E-cadherin (#610181) and N-cadherin (#610920) were purchased from BD Biosciences (San Jose, CA, USA). The antibody recognizing p-YAP (Tyr357) (ab62751) was purchased from Abcam (Cambridge, United Kingdom). Antibodies recognizing Vimentin (sc-32322) was purchased from Santa Cruz biotechnology (Dallas, TX, USA). V5 Tag (#A190-120A), Alexa Fluor 488 donkey anti-mouse IgG and Alexa Fluor 568 goat anti-rabbit IgG were purchased from Thermo Fisher Scientific (Cleveland, OH, USA). Saracatinib (#S1006) and PP2 (#S7008) were purchased from Selleckchem (Houston, TX, USA). G418 was purchased from Biosesang (Gyeonggi-do, South Korea).

### Western blot analysis

Total cell lysates were prepared and immunoblotting was performed as previously reported (Kim et al., 2019).

### Real-time quantitative reverse transcription polymerase chain reaction (RT-qPCR) and bioinformatic analysis

TRIzol reagent (Invitrogen, Carlsbad, CA, USA) was used to extract total RNA in accordance with the manufacturer’s instructions. RNA-sequencing was performed by Macrogen, Inc. (Seoul, South Korea). Hippo signaling-related genes used for producing a heatmap include Gene Ontology biological process (GOBP) Hippo signaling gene set (https://www.gsea-msigdb.org/gsea) and a YAP/TAZ transcriptional target signature of 22 genes (Wang Y. et al., 2018). One microgram of total RNA was reverse-transcribed to cDNA by Maxime RT-PreMix Kit (Intron biotechnology, Seongnam, South Korea). Real-time PCR was performed with the MiniOpticon real-time PCR analysis instrument (Bio-Rad, Hercules, CA, USA) by using IQ Sybr Green SuperMix (Bio-Rad). The 18S mRNA value was used to normalize the target gene’s mRNA level. The following primer sets were used for the amplification of targets: Human ERα36 (forward: 5′-GAC​AGG​AAC​CAG​GGA​AAA-3′, reverse: 5′- TCT​ACA​TGT​GAG​ATA​CCA​GA-3′), human YAP (forward: 5′-ACG​TTC​ATC​TGG​GAC​AGC​AT-3′, reverse: 5′-GTT​GGG​AGA​TGG​CAA​AGA​CA-3′), human CTGF (forward: 5′-CCA​ATG​ACA​ACG​CCT​CCT​G-3′, reverse: 5′-TGG​TGC​AGC​CAG​AAA​GCT​C-3′), human CYR61 (forward: 5′- AGC​CTC​GCA​TCC​TAT​ACA​ACC-3′, reverse: 5′-TTC​TTT​CAC​AAG​GCG​GCA​CTC-3′), human 18S rRNA (forward: 5′-GTA​ACC​CGT​TGA​ACC​CCA​TT -3′, reverse: 5′- CCA​TCC​AAT​CGG​TAG​TAG​CG -3′).

### Transfection

ERα36-MCF-7 cells were constructed as previously described by Wang and others (Wang et al., 2005; Wang et al., 2006), and hER-α36 expression vector was kindly provided by Dr. Zhao-Yi Wang (Creighton University Medical School, USA). MCF-7 cells were plated at a density of 1 × 105 cells/well in a 24-well plate overnight and the cells were transfected with either a hER-α36 expression vector or empty pcDNA3.1 vector as control vector driven by the CMV promotor using Lipofectamine 2000 (Thermo Fisher Scientific). Transfected cells were replated 48 h after transfection and selected for several weeks using G418 (800 μg/ml).

For immunoprecipitation assay, ERα36-V5-His overexpressing MCF-7 cells were also established by retrovirus infection system: MCF-7 cells were exposed to the viral soup obtained by introducing MSCV-ERα36-V5-His vector to Phoenix-AMPHO cells with 4 μg/ml polybrene (Santa Cruz Biotechnology, Dallas, TX, USA). Infected cells were isolated by sorting GFP-labeled cells with FACSAria II cell sorter (BD Biosciences).

YAP knockout MCF-7-ERα36 cells were constructed by CRISPR-Cas9 gene editing as previously reported (Park et al., 2021). The plasmid DNA U6-gRNA/CMV-Cas9-RFP plasmid for YAP (HS0000121498) and CRISPR universal negative control plasmid were purchased from Sigma (San Luis, MO, USA).

### Luciferase reporter gene assay

Using Lipofectamine 2000 (Thermo Fisher Scientific), the indicated cells were transfected with the 8×GTIIC-luciferase vector (#34615, Addgene) and the Renilla luciferase-encoding pRL-TK plasmid. The firefly and Renilla luciferase activities were determined by the Dual-Luciferase Reporter Assay System (Promega, Madison, WA, USA) using a luminometer (Centro LB 960, Berthold Technologies, Bad Wildbad, Germany). The promoter-driven firefly luciferase activity was normalized to the pRL-TK (Renilla) luciferase activity to determine the relative luciferase activities.

### Immunocytochemistry

Immunocytochemistry was performed as previously described (Park et al., 2021). Briefly, MCF-7 and MCF-7-ERα36 cells were cultured overnight on the coverslips, fixed with 4% paraformaldehyde for 20 min at room temperature, and incubated with 0.1% Triton X-100 for 15 min. The fixed cells were blocked with 10% horse serum for 1 h and incubated with the indicated primary antibody (1:200), followed by the incubation with fluorophore-conjugated secondary antibody (1:1000). Finally, the coverslips were washed with PBS and then mounted with ProLong Gold Antifade reagent with 4’,6-diamidino-2-phenylindole (DAPI; Invitrogen, Carlsbad, CA, USA). Images were obtained using confocal microscope (Leica TCS SP8 MP, Leica Microsystems, Wetzlar, Germany).

### Real-time cell proliferation monitoring

Cells were plated at a density of 3 × 103 in 96-well plates, and the phase confluence of the cells was monitored every 4 h using an IncuCyte S3 Live Cell Analysis System (Sartorius, Ann Arbor, MI, USA). For 3-(4,5-dimethylthiazol-2-yl)-2,5-diphenyltetrazolium bromide (MTT) assay, MCF-7-CTRL and MCF-7-ERα36 cell lines were cultured overnight on a 96-well plate at 4 × 103 cells/well, and then verteporfin was treated in a concentration-dependent manner. After 24 h, cells were exposed to 1 mg/ml MTT solution for 2 h, and 200 μL dimethyl sulfoxide (DMSO) was added after removing the MTT solution. The absorbance was measured at a wavelength of 540 nm using a SpectraMax i3x Multi-mode microplate reader (Molecular Devices, CA, USA).

### Three-dimensional tumor spheroid assay

Cells were dispensed into an Ultra-low attachment (ULA) plate (#7007, Corning Incorporated, Corning, NY, USA) at 2 × 103 cells/well and centrifuged at 300 g for 5 min. The tumor sphere images were captured using the Incucyte S3 Live Cell Analysis System at 168 h and the diameters were measured. The volume was calculated using the formula (length×width2)×π/6.

### Immunoprecipitation assay

To observe the binding of V5-labeled ERα36 to FLAG-YAP (kindly donated from Dr. Kwang Youl Lee, Chonnam National University, Gwangju, South Korea), 2 μg of anti-FLAG antibody was added to 500 μg of cell lysates and reacted at 4°C for 16 h. Antigen-Antibody conjugate was captured with protein G agarose beads (Sigma) at 4°C for 1 h, then precipitated and boiled in a heat block for 5 min. The extracted protein was observed by immunoblotting.

### Statistical analysis

The values were presented as means ± SD. Unpaired Student t test or one-way analysis of variance (ANOVA) was used to assess statistical significance. Results were considered significant when *p* < 0.05 (*, *p* < 0.05; **, *p* < 0.01; ***, *p* < 0.005; ****, *p* < 0.001).

## Results

### Overexpression of ERα36 in MCF-7 cells promotes cell proliferation, 3D spheroid formation and EMT phenotypes

**FIGURE 1:**
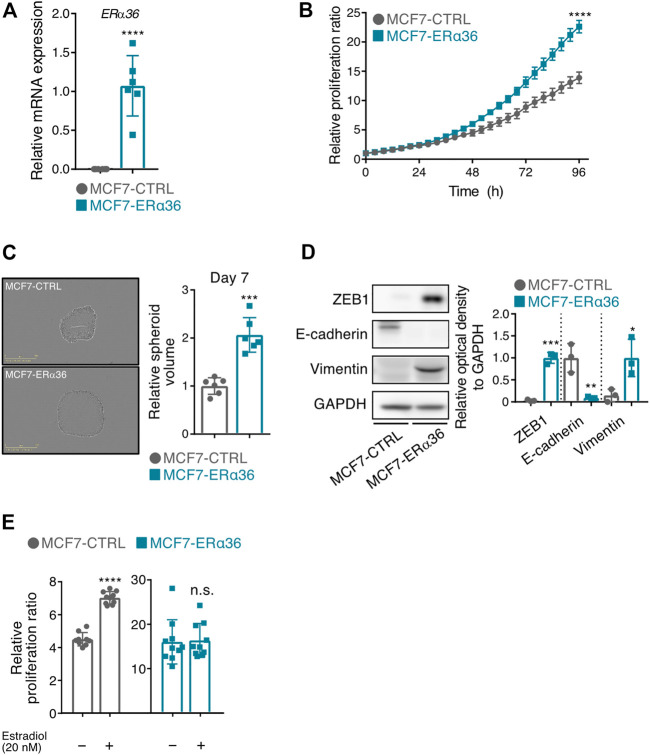
Overexpression of ERα36 in MCF-7 cells promotes cell proliferation, 3D spheroid formation and EMT-phenotypic change. **(A)** The mRNA level of ERα36 was verified by RT-qPCR in MCF-7-CTRL and MCF-7-ERα36 cell. **(B)** Proliferation of the indicated cells was monitored by using a real-time cell imaging system, and the relative ratio was calculated by setting the phase confluence at 0 h to 1 (n = 6). **(C)** Estimation of 3D-spheroid formations by using a real-time cell imaging system. **(D)** The expression levels of EMT-related proteins were determined in MCF-7-CTRL and MCF-7-ERα36 cells (left). The relative optical density of those proteins was indicated (right). **(E)** Proliferation of the indicated cells was determined in the presence or absence of 17-beta-estradiol (20 nM) under 10% charcoal-stripped FBS condition. Each phase confluence at 72 h was normalized to that at 0 h. All statistical significance of the differences was determined by unpaired two-tailed Student *t* test. n. s., not significant; *,*p* < 0.05; **,*p* < 0.01; ***,*p* < 0.005; ****,*p* < 0.001 significant as compared with MCF7-CTRL cells **(A–D)** or vehicle-treated cells **(E)**. An abbreviation is as follows: CTRL, control vector.

To investigate the function of ERα36, we established a cell line that stably overexpresses ERα36 in MCF-7 cells, an ER-positive luminal A subtype among human breast cancer cells (Figure 1A). Overexpression of ERα36 in the MCF-7 cells significantly increased cell proliferation under the 10% FBS condition, as is consistent with the notion that ERα36 can lead to proliferation in several breast cancer cell lines (Mahboobifard et al., 2021) (Figure 1B). Next, we compared the 3D-sphere-forming ability on the seventh day after cells were dispensed onto a ULA plate. The volume of 3D spheres of the MCF-7-ERα36 cells was prominently elevated relative to the MCF-7-CTRL cells (Figure 1C). In addition, the MCF-7-ERα36 cells became, in their morphology, more like mesenchymal cell types than did the MCF-7-CTRL cells. As shown in Figure 1D, the protein expression of several EMT markers including E-cadherin, ZEB1 and Vimentin was significantly changed by overexpression of ERα36. Intriguingly, the estrogen dependency for proliferation was significantly reduced by overexpression of ERα36 in MCF-7 cells under the 10% charcoal-stripped FBS condition (Figure 1E). These data suggest that ERα36 overexpression could induce aggressive phenotypic changes in MCF-7 cells.

### The potential association between ERα36 and YAP

**FIGURE 2:**
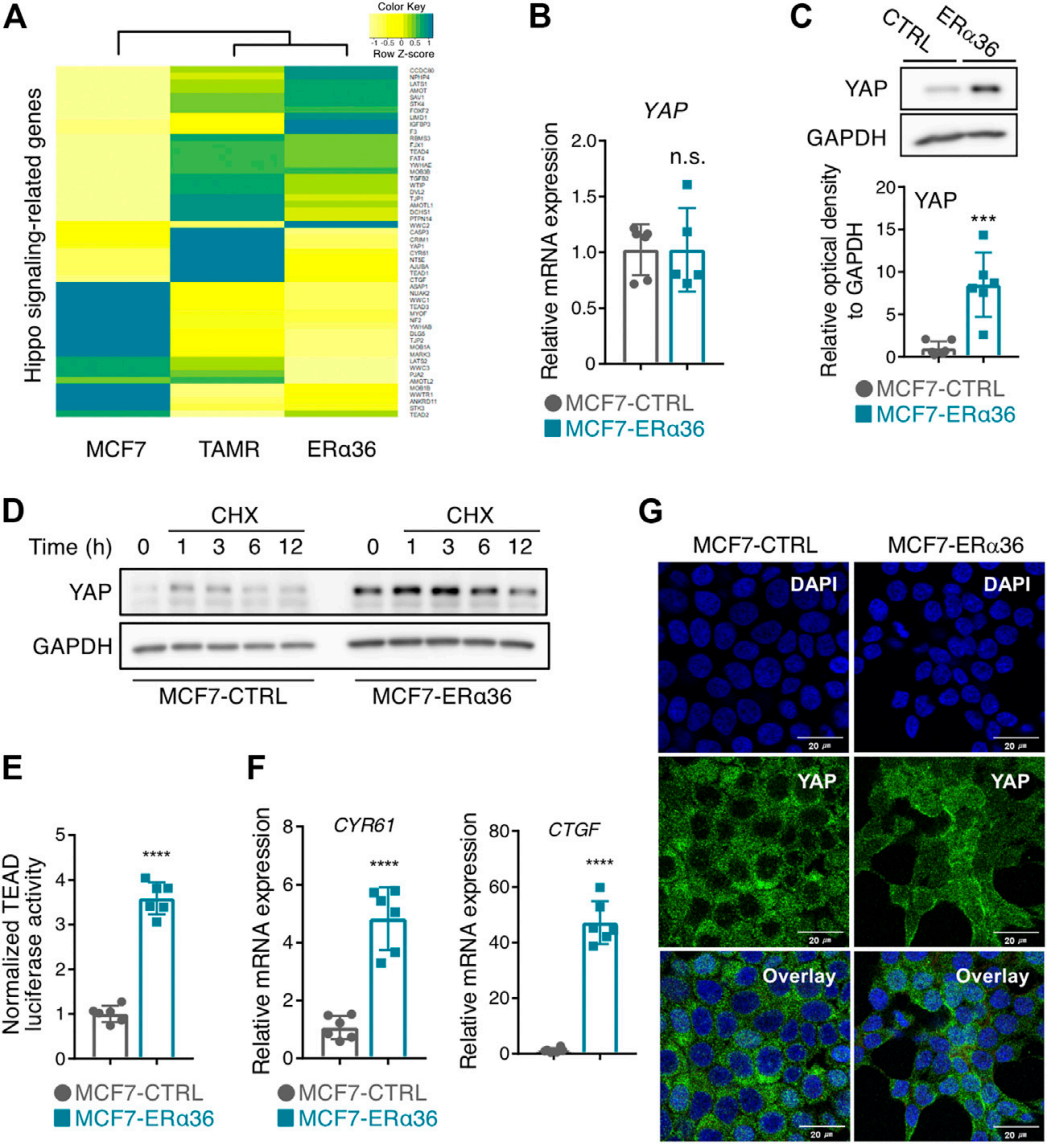
Activation of YAP by overexpression of ERα36 in MCF-7 cells. **(A)** Hippo signaling-related gene expression derived from RNA-sequencing analysis of MCF-7, TAMR-MCF-7, and MCF-7-ERα36 cells. **(B)** The mRNA levels of *YAP* in MCF-7-CTRL and MCF-7-ERα36 cells. **(C)** The elevated YAP protein expression in MCF-7-ERα36 cells compared to MCF-7 control cells (up) and the relative optical density of YAP (down). **(D)** The half-life of YAP protein was analyzed in a time-dependent condition in the presence of cycloheximide (100 μM). **(E)** YAP/TAZ-responsive TEAD reporter activities in the indicated cells. **(F)** The mRNA levels of *CTGF* and *CYR61* in the indicated cells. **(G)** The elevated nuclear expression of YAP in the indicated cells analyzed by immunocytochemical staining. All statistical significance of the differences was determined by unpaired two-tailed Student *t* test. n. s., not significant; *, *p* < 0.05; ****, *p* < 0.001 significant as compared with MCF7-CTRL cells. CTRL, control vector. CHX, cycloheximide.

Higher ERα36 expression in breast cancer patients receiving tamoxifen treatment has been linked to worse survival in cohort studies (Shi et al., 2009). Treatment with tamoxifen also increases ERα36-positive breast cancer cell populations (Shi et al., 2009; Wang Q. et al., 2018). In order to identify novel genes associated with ERα36-related tamoxifen resistance, we analyzed the transcriptomes in MCF-7, tamoxifen-resistant MCF-7 (TAMR-MCF-7), and ERα36-MCF-7 cells. The data obtained revealed that the expression pattern of genes in the Hippo signaling pathway is very similar between TAMR-MCF-7 and MCF-7-ERα36 cells (Figure 2A). Because YAP, a downstream target gene of the Hippo pathway, is known to be related to tamoxifen sensitivity (Kim et al., 2022), we hypothesized that ERα36 might be involved in YAP activity in driving proliferation and promoting EMT phenotypes in MCF-7 cells.

Whereas the mRNA level of YAP in the MCF-7-ERα36 cells was comparable to that in the MCF-7 cells (Figure 2B), its protein level was highly increased in the MCF-7-ERα36 cells (Figure 2C). These results raise the possibility that YAP protein expression is post-translationally regulated by ERα36. Indeed, overexpression of ERα36 prolonged the half-life of YAP after treatment with cycloheximide, a protein synthesis inhibitor (Figure 2D). We next determined whether upregulated protein expression of YAP can lead to its increased activities in MCF-7-ERα36 cells. As shown in Figure 2E, a TEAD-reporter luciferase assay confirmed that YAP/TAZ-dependent transcription activity was highly increased in the MCF-7-ERα36 cells. Moreover, the mRNA levels of CYR61 and CTGF, representative downstream target genes of YAP, also were elevated in the same cells (Figure 2F). Immunocytochemistry results revealed that ERα36 overexpression induced nuclear localization of YAP (Figure 2G). These data demonstrate that ERα36 overexpression in MCF-7 cells can activate YAP by upregulation of its protein stability.

### Inhibition of YAP reverses ERα36-induced cell proliferation, 3D spheroid formation and EMT marker expression

To investigate the role of YAP in the cell survival of ERα36-overexpressing MCF-7 cells, we used verteporfin, a small-molecule inhibitor of YAP, to block YAP-TEAD interaction (Liu-Chittenden et al., 2012). MTT assay results showed MCF-7-ERα36 cells’ higher sensitivity to verteporfin (IC_50_ value: 8.6 μM in MCF-7 vs 3.0 μM in MCF-7-ERα36 cells), indicating that they are more dependent on YAP activity for survival than are MCF-7-CTRL cells.

**FIGURE 3:**
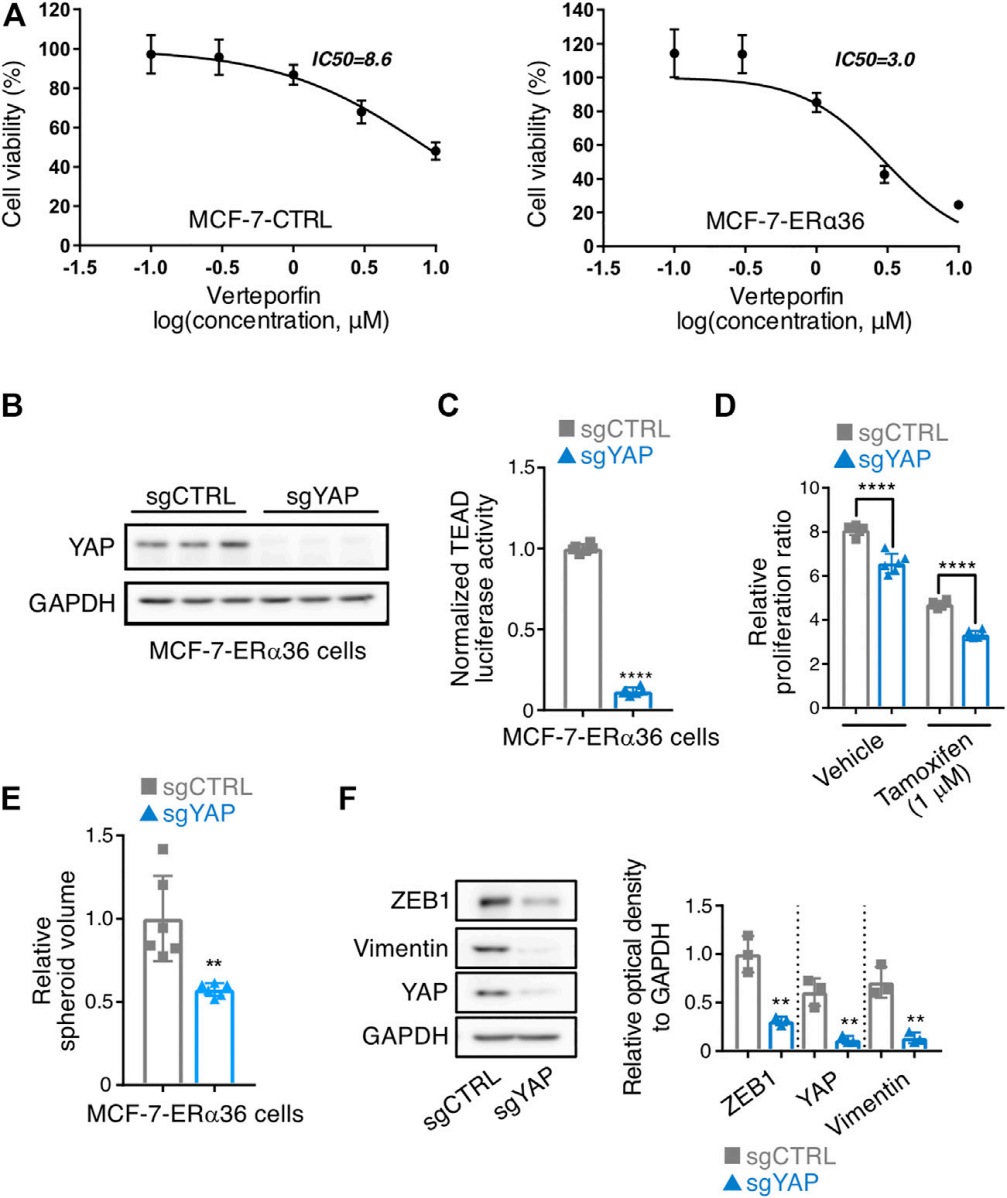
Inhibition of YAP reverses ERα36-induced cell proliferation, 3D spheroid formation and EMT marker expression. **(A)** MTT analysis in MCF-7-CTRL and MCF-7-ERα36 cells after exposure to verteporfin (n = 6). **(B)** Silencing of YAP by CRISPR-Cas9-gRNA. Expression of YAP was determined by Western blot analysis to confirm its knockout. **(C)** YAP/TAZ-responsive TEAD reporter activities in the indicated cells. **(D)** Effect of YAP knockout on the cell proliferation and responsiveness to tamoxifen in MCF-7-ERα36 cells. Cells were treated with tamoxifen for 102 h. **(E)** Inhibition of 3D spheroid formations estimated by using a real-time cell imaging system in the indicated cells. **(F)** Expression of EMT-related proteins in the indicated cells (left) and the relative optical density of them (right). All statistical significance of the differences was determined by unpaired two-tailed Student *t* test. **, *p* < 0.01; ****, *p* < 0.001 significant as compared with ERα36 sgCTRL cells. An abbreviation is as follows: sgCTRL, single guide RNA used as a negative control.

We then established YAP-knockout MCF-7-ERα36 cells (sgYAP) using the CRISPR system (Figure 3B). A TEAD luciferase reporter assay confirmed that YAP-dependent transcription had been diminished in sgYAP MCF-7-ERα36 cells (Figure 3C). Interestingly, YAP knockout in MCF-7-ERα36 cells reduced cell proliferation, and this cell type was more sensitive to tamoxifen treatment (Figure 3D). In addition, the 3D spheroid volume and mesenchymal cell marker expression were decreased by YAP knockout in MCF-7-ERα36 cells (Figures 3E,F). These data suggest that YAP promotes the aggressive phenotypes acquired by ERα36 overexpression.

### Role of Src in ERα36-mediated YAP activation

**FIGURE 4:**
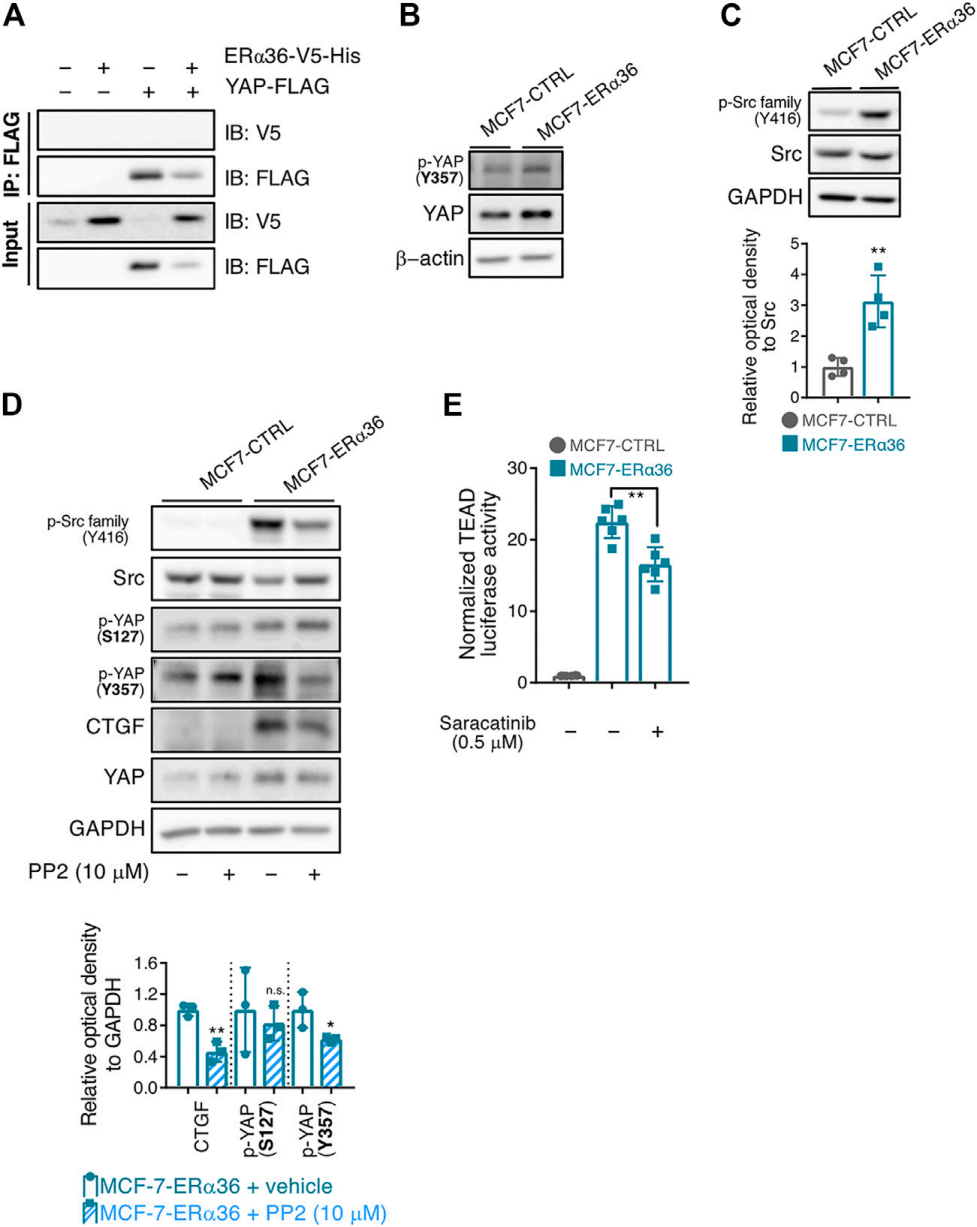
Role of Src in the ERα36-induced YAP activation. **(A)** Immunoprecipitation and immunoblotting of V5 and FLAG proteins in MCF-7-MSCV-CTRL and MCF-7-MSCV-ERα36-V5-His cells. **(B)** Increased expression of p-YAP (Tyr357) protein in MCF-7-ERα36 cells compared to MCF-7-CTRL. **(C)** Expression of p-Src family (Tyr416) and Src in the indicated cells (up) and the relative optical density of p-Src protein (down). **(D)** Effect of PP2 (10 μM, 24 h), a Src family kinase inhibitor on the expression level of each indicated protein in MCF-7-CTRL and MCF-7-ERα36 cells (up) and the relative optical density of them (down). **(E)** TEAD reporter activities in MCF-7-ERα36 cells treated with saracatinib, a Src inhibitor for 24 h. All statistical significance of the differences was determined by unpaired two-tailed Student *t* test. n. s., not significant; *, *p* < 0.05; **, *p* < 0.01; significant as compared with MCF7-CTRL cells **(C)** or vehicle-treated cells **(D–E)**.

To clarify the detailed mechanism of how ERα36 can regulate the transcriptional activity of YAP, we first assessed whether ERα36 could directly bind to YAP. To that end, we transfected MCF-7 cells overexpressing ERα36-V5-His with FLAG-tagged YAP plasmid and performed FLAG immunoprecipitation to detect ERα36-V5. However, YAP did not bind to ERα36 (Figure 4A). We then explored whether YAP could be activated by indirect signaling pathway(s) in response to ERα36.

Note first that the protein expression and activity of YAP can be controlled by its phosphorylation. Specifically, phosphorylation of YAP at Ser127 promotes its cytoplasmic retention and degradation by 14-3-3ζ-mediated ubiquitination, thereby suppressing its transcriptional activity (Zanconato et al., 2016). In contrast, phosphorylation of Tyr357 and other tyrosine residues enhances nuclear translocation of YAP and increases its ability to stimulate transcription in the nucleus (Dasgupta and McCollum, 2019). Meanwhile, Src and Src-family kinases belong to the signal transduction pathways downstream of both ERα66 and ERα36, and are known to directly interact with ERα36 (Chu et al., 2007; Zhang et al., 2011; Pagano et al., 2020). In addition, Src can mediate the phosphorylation of YAP and promote its protein stability (Rosenbluh et al., 2012; Li et al., 2016; Byun et al., 2017; Smoot et al., 2018; Lamar et al., 2019). Src-family kinase especially increases the amounts of Tyr357-phosphorylated YAP, a representative nuclear form of YAP, regardless of LATS activity (Sugihara et al., 2018).

Surprisingly, ERα36 overexpression in MCF-7 cells promoted Tyr357 phosphorylation of YAP (an active form of YAP) (Figure 4B). Based on our result that Tyr416 phosphorylation of the Src family (an active form of Src) was also increased in MCF-7-ERα36 cells (Figure 4C), we evaluated the involvement of Src in ERα36-mediated phosphorylation of YAP at Tyr357. As expected, treatment with PP2, a Src-family kinase inhibitor, decreased the protein expression of both p-YAP (Tyr357) and CTGF (Figure 4D). We also found that saracatinib, the potent Src kinase inhibitor, reduces TEAD-reporter luciferase activity in MCF-7-ERα36 cells (Figure 4E). Taken together, our data indicate that Src activation plays a critical role in ERα36-induced YAP activation in MCF-7 cells.

## Discussion

Although the 5-year relative survival rate for female breast cancer is relatively higher (90%), the 5-year survival rate for breast cancer patients with distant metastasis drops precipitously to 27%, thus urgently requiring effective treatment for resistant and metastatic breast cancer (Siegel et al., 2021). The classification of breast cancer based on the presence or absence of hormone receptors determines the treatment strategies (Waks and Winer, 2019). To provide a better therapeutic option to breast cancer patients, there have been many attempts to identify unknown target molecules and novel gene-expression patterns in breast cancer (Bianchini et al., 2022). In the current study, we focused on the functions and signaling pathways of ERα36, a variant of ERα, in ER-positive breast cancer cells.

In tamoxifen-resistant or ER-negative breast cancer cells and tissues, ERα36 expression was increased and it plays an important role in tumor growth, progression, transformation, and metastasis (Zhang X. et al., 2012; Li et al., 2013; Su et al., 2014; Maczis et al., 2018; Maczis et al., 2018). Knocking down ERα36 resulted in decreased migration and invasion as well as increased paclitaxel sensitivities in MDA‐MB‐231 cells (Zhang J. et al., 2012). In particular, tamoxifen could serve as an agonist of ERα36 and enhances the stemness and metastasis of breast cancer cells *via* enhancing aldehyde dehydrogenase 1A1 and cause breast cancer cells to proliferate, invade, and metastasize (Wang Q. et al., 2018; Pagano et al., 2020).

We demonstrated that ERα36 promotes cell proliferation, three-dimensional sphere formation, and EMT of MCF-7 cells, thus confirming its roles in malignant transformation of breast cancer. When we compared the transcriptome profiles of MCF-7, TAMR-MCF-7, and MCF-7-ERα36 cells, the expression patterns of genes involved in the Hippo pathway were highly similar between the TAMR-MCF-7 and MCF-7-ERα36 cells, whereas the pattern in MCF-7 cells was distinctly different. Moreover, the protein levels of YAP, a downstream effector of the Hippo pathway and its target genes such as CYR61 and CTGF, were significantly increased by ERα36 overexpression in MCF-7 cells.

YAP has attracted considerable interest due to its role in cell proliferation, tumorigenesis, metastasis, and EMT in the tumor microenvironment (Zanconato et al., 2016; Cheng et al., 2020). Previous reports have suggested that YAP is more actively related to the proliferation of aggressive types of breast cancer than ERα-positive breast cancer. YAP has been shown to inhibit ERα-positive tumor growth by disrupting ERα/TEAD interaction while promoting proliferation of MDA-MB-231, a TNBC cell line (Li et al., 2022). Similarly, YAP expression is positively correlated with cell proliferation in the ER-negative sub-group but inversely correlated in the ER-positive sub-group (Lehn et al., 2014). Kim et al. recently reported that expression of YAP, CTGF, and CYR61 is elevated in recurrent breast cancer tissues after tamoxifen treatment; they also showed that YAP overexpression leads to tamoxifen-resistance and downregulation of ERα (Kim et al., 2021), as is consonant with the notion that expression of ERα is decreased in TNBC cell lines. However, there is still a lack of research on the relationship between ERα36 and YAP. Herein we have presented, for the first time, the direct link between ERα36 and YAP. ERα36 increased the stability and the nuclear distribution of YAP, and the deletion of YAP in MCF-7-ERα36 reversed the acquired aggressive phenotypes induced by ERα36.

Our results exemplified that ERα36-induced Src activation regulates the stability and location of YAP *via* phosphorylation of its Tyr357 residue in ERα36-MCF-7 cells. To clarify the YAP regulatory mechanism by ERα36, we investigated whether ERα36 directly binds to YAP to control its activity. However, no direct interaction between ERα36 and YAP was observed (Figure 4A). The sub-signal transduction pathways of ERα36 include MAPK/ERK, c-Jun N-terminal kinase (JNK), and the AKT/protein kinase B pathway (Wang et al., 2006; Lin et al., 2009; Lin et al., 2010; Zhang et al., 2012; Zhang et al., 2012). ERα36 can also physically interact with the EGFR/Src/Shc complex and stimulate rapid non-genomic signaling (Zhang et al., 2011). Src and Src-family kinases directly or indirectly phosphorylate YAP/TAZ to increase protein stability and transcriptional activity (Rosenbluh et al., 2012; Li et al., 2016; Byun et al., 2017; Smoot et al., 2018; Lamar et al., 2019). The two main proposed mechanisms for Src-family-mediated YAP activation are: 1) Hippo independent pathway - direct phosphorylation at Y341, Y357, and Y394, which causes YAP to translocate to the nucleus; 2) Direct inhibition of Hippo kinases or activation of pathways such as MAPK, PI3K and Rho, which suppresses Hippo kinases (Hsu et al., 2020). We confirmed that ERα36 overexpression significantly elevates p-LATS activity in MCF-7 cells (data not shown) while the protein level of p-YAP (Ser127) remained unchanged by ERα36 overexpression. These results indicate that Hippo kinases might not be involved in ERα36-mediated YAP activation. Meanwhile, we showed that the expression levels of both p-YAP (Tyr357) and the p-Src family (Tyr416) were increased in MCF-7-ERα36 cells relative to control cells. PP2, an Src-family kinase inhibitor, diminished Tyr357 phosphorylation of YAP in MCF-7-ERα36 cells and reduced the expression level of CTGF, a representative target gene of YAP. Therefore, ERα36-induced Src activation may regulate the stability and location of YAP *via* phosphorylation of its Tyr357 residue in breast cancer cells.

In summary, we identified a new signal transduction mechanism of ERα36 that focuses on YAP-induced proliferation, EMT, and 3D spheroids in MCF-7 cells. Our results shed some light on the role of YAP as activated by ERα36, the expression of which was upregulated in tamoxifen-resistant and TNBC cells. Our results suggest that targeting of the Src-YAP axis could be a promising strategy, especially for those patients whose tumors show higher ERα36 expression. However, there still remain questions as to whether the ERα36-Src-YAP axis universally applies to other aggressive breast cancer cell types such as TNBC cells or *in vivo* animal models. Future studies using clinically relevant animal models and patients’ data are needed in order to elucidate the potential roles of ERα36 and the YAP axis in aggressive breast cancers.

## Data Availability

The original contributions presented in the study are included in the article/supplementary material, further inquiries can be directed to the corresponding author.
